# Protective Immunity against Lethal *F. tularensis holarctica* LVS Provided by Vaccination with Selected Novel CD8^+^ T Cell Epitopes

**DOI:** 10.1371/journal.pone.0085215

**Published:** 2014-01-06

**Authors:** Shahar Rotem, Ofer Cohen, Erez Bar-Haim, Liat Bar-On, Sharon Ehrlich, Avigdor Shafferman

**Affiliations:** Department of Biochemistry and Molecular Genetics, Israel Institute for Biological Research, Ness-Ziona, Israel; Albany Medical College, United States of America

## Abstract

Recently we described an unbiased bacterial whole-genome immunoinformatic analysis aimed at selection of potential CTL epitopes located in “hotspots” of predicted MHC-I binders. Applying this approach to the proteome of the facultative intra-cellular pathogen *Francisella tularensis* resulted in identification of 170 novel CTL epitopes, several of which were shown to elicit highly robust T cell responses. Here we demonstrate that by DNA immunization using a short DNA fragment expressing six of the most prominent identified CTL epitopes a potent and specific CD8+ T cell responses is being induced, to all encoded epitopes, a response not observed in control mice immunized with the DNA vector alone Moreover, this CTL-specific mediated immune response prevented disease development, allowed for a rapid clearance of the bacterial infection and provided complete protection against lethal challenge (10LD_50_) with *F. tularensis holarctica* Live Vaccine Strain (LVS) (a total to 30 of 30 immunized mice survived the challenge while all control DNA vector immunized mice succumbed). Furthermore, and in accordance with these results, CD8 deficient mice could not be protected from lethal challenge after immunization with the CTL-polyepitope. Vaccination with the DNA poly-epitope construct could even protect mice (8/10) against the more demanding pulmonary lethal challenge of LVS. Our approach provides a proof-of-principle for selecting and generating a multi-epitpoe CD8 T cell-stimulating vaccine against a model intracellular bacterium.

## Introduction

Many virulent bacteria can grow intracellularly in infected hosts and exploit this ability as a key pathogenic and immune evasion strategy. Efficient protection against such microorganisms is critically dependent on the development of cellular immunity, particularly the CD8+ T cell mediated response. Consequently, the identification of immunogenic and protective CD8+ T cell epitopes presents a significant challenge in the development of efficacious vaccines against intracellular bacterial pathogens [1].

*Francisella tularensis*, the causative agent of tularemia, is one of the most highly infectious facultative intracellular gram-negative bacterial pathogens documented. Two main subspecies are pathogenic in humans. (I) *F. tularensis* subsp. *tularensis* (also known as type A), which is predominantly found in North America, is highly infectious and virulent and causes a fulminant and often fatal disease after inhalational exposure to as few as 10 microorganisms. (II) *F. tularensis* subsp. *holarctica* (type B) is found throughout Europe and Eurasia and causes a milder disease in humans [2]. *F. tularensis* has been classified by the Center for Disease Control and Prevention (CDC) as a Category A bioterrorism agent [3], [4].

*F. tularensis* replicates intracellularly within both human and murine macrophages, thereby evading the host immune system [5]–[7]. Acquired host resistance against *F. tularensis* involves both the humoral and cell-mediated immune responses. Both CD4+ and CD8+ T cells are sufficient to resolve an LVS primary infection, as mice depleted of either population individually clear the bacteria from the tissues [8]–[10]. For secondary LVS IP challenges, either CD4+ or CD8+T cell subsets are sufficient for survival and clearance of an LVS infection. In contrast, full resistance to virulent *F. tularensis* pulmonary secondary challenges of vaccinated mice requires both CD4+ and CD8+ T cells, as depletion of either T cell subset significantly reduces survival [11]–[13].

A live vaccine was developed by soviet scientists, which was an attenuated *holarctica* strain, named strain 15. This vaccine was highly effective at preventing tularemia caused by subsp. *holarctica* [14]. USA developed a phenotypically more defined live vaccine strain (LVS) derived from strain 15 [15]. LVS which is attenuated in humans but pathogenic in mice, [16], [17] was tested on human volunteers in the past, with variable efficacy against intradermal or aerosol challenge which was dependent on challenge dose and the time interval between vaccination and exposure [18]–[20]. These, as well as other regulatory concerns, prompted the search for novel vaccines against tularemia. Strategies for the development of a new generation of tularemia vaccines include the exploration of different live and killed attenuated strains and the identification of bacterial components that can act as subunit vaccines [18]–[25].

We have recently developed and established a novel approach for the unbiased whole-genome selection of putative CD8+ T cell epitopes [26], [27]. This approach was based on the mapping of immunological ‘hotspots’ (cluster regions of 8–25 amino acids harboring consecutive predicted MHC class I binders) and selection of highly dense clusters for further experimental evaluation. With this approach, out of 1640 screened peptides, we identified 170 novel *F. tularensis* CTL epitopes with predicted affinities of 5–988 nM. Several of these epitopes were shown to elicit highly robust T cell responses. Interestingly, these epitopes are mostly located within hypothetical proteins, and none of these proteins was previously described to be immunogenic [26], [27].

In view of the importance of CD8+ T cells in conferring protective response against intracellular pathogens, we sought to examine the ability of the newly identified immunodominant CD8 epitopes to induce a CD8 dependent anti-*Francisella* protective immunity, in the mouse model. We demonstrate that a DNA construct expressing a short poly-epitope composed of the six most prominent CTL epitopes can elicit in mice a specific CD8+ T cell response that is sufficient to provide effective protection against lethal systemic as well as a lethal pulmonary *F. tularensis* LVS challenge.

## Materials and Methods

### Ethics Statement

This study was carried out in strict accordance with the recommendation in the Guide for the Care and Use of Laboratory Animals of the National Institute of Health. All experiments were approved by the animal care and use committee at the Israel Institute for Biological Research (Permit Numbers: IACUC-IIBR M-52-2011, IACUC-IIBR M-16-2012, IACUC-IIBR M-35-2012 and IACUC-IIBR M-68-2012) and were carried out in accordance with the Animal Welfare Act.

During the experiments, humane endpoints were used for vaccinated and infected mice. Mice were monitored daily for lack of motility, loss of contact sensitivity or loss of the righting reflex. Mice were euthanized by cervical dislocation.

### Animals

Female C57BL/6J, CD4^−/−^ (B6.129S2-*Cd4^tm1Mak^*/J) and CD8^−/−^ (B6.129S2-*Cd8a^tm1Mak^*/J) mice (6 to 8 weeks old) were obtained from Jackson Laboratories (Bar Harbor, Maine, US) and randomly assigned into cages in groups of 6 to 10 animals. The mice were allowed free access to water and rodent diet (Harlan, Israel). Note that all mice used in this report belong to the H2^b^ allelic restriction.

### Preparation of Bacteria and Animal Challenge

*F. tularensis* subsp. *holarctica,* strain LVS (ATCC 29684), and *F. tularensis* subsp. *tularensis,* strain Schu S4 were used in the challenge experiments. All Schu S4 manipulations were carried out in a Class 3 microbiological safety conditions. The median lethal dose (LD_50_) of untreated animals was 10^2^ CFU and 10^3^ CFU for the LVS *intra-peritoneal (i.p.*) and *intra-nasal (i.n.*) routes of exposure, respectively. The median lethal dose (LD_50_) of untreated animals for the Schu S4 strain was 1 CFU for the *i.p.* route of exposure. Bacterial glycerol stocks that had been stored at –80°C were streaked onto cysteine heart agar (CHA) (Becton Dickinson, France) and incubated for 1–2 days at 37°C. Bacterial cultures were grown to mid-log phase (optical density of 0.1–0.2 at 660 nm) at 37°C in TSBC (TSB Difco, supplemented with 0.1% cysteine) for LVS and PPB (Bacto proteose peptone, Difco; supplemented with 1% Glucose, 0.5% NaCl and 0.05% cysteine, Sigma) for the Schu S4 strain at 37°C [28], [29]. The bacteria were washed and re-suspended in PBS at the desired concentrations. Animals were challenged with 10 LD_50_ of LVS (for *i.p*. 1000 CFU in 200 µl*;* for *i.n.*, 10,000 CFU in 25 µl) or with 10 LD_50_ of the fully virulent Schu S4 (10 CFU in 200 µl *i.p.*). Challenges of vaccinated mice were performed 14 days after the last immunization. LVS inactivation was performed by incubating 5×10^9^ CFU/ml of LVS with 0.4% formalin in PBS at room temperature overnight, subsequently the bacteria were washed extensively with PBS.

### Multi-epitope-based DNA Vaccine Construct (DNA-PolyEp)

The DNA-PolyEp construct was designed to include six of the strongest H-2^b^ restricted epitopes (an allelic restriction of C57BL/6 mice) that were identified in our previous *F. tularensis* whole-genome scan [26]. The CTL epitopes (1 to 6, Table 1) are located within six different proteins, including Trk (FTL_1708_13–23_ (epitope 3) and five other hypothetical proteins: FTL_1916_400–410_ (epitope 1), FTL_0966_126–133_ (epitope 2), FTL_0283_95–104_ (epitope 4), FTL_1101_64–74_ (epitope 5), and FTL_1673_308–318_ (epitope 6). A synthetic DNA fragment was designed to express codon-optimized epitopes 1 to 6 in tandem (see Table 1). This fragment (186 bp) was synthesized (GenScript, NJ, USA) and cloned into the pCI plasmid DNA vaccine eukaryotic expression vector (4006 bp) (Promega, USA), routinely used for DNA vaccination, to generate DNA-PolyEp. The sequence of the construct was verified. The original pCI vector was used as a control vaccine.

**Table 1:** Induction of T cell response to selected CTL epitopes following LVS or DNA-PolyEp immunization.

	No. of IFNγ secreting cells/10^6^ splenocytes^d^		
Stimulating antigensa,b	Immunization^c^		
	LVS	DNA-PolyEp (1–6)^e^	DNA Vector (control pCI)
(1) SYWSYFSFFSL	53±6	12±1	<5
(2) IAMTLLTM	71±7	26±4	<5
(3) LMFLSLTMLSP	66±3	20±5	<5
(4) YSLLTAYIFM	54±6	10±3	<5
(5) SFFNYFKYMGM	67±9	10±2	<5
(6) IILYVPMSLSM	54±6	42±6	<5
Peptide mix of (1)–(6)	95±6	83±1	<5
Positive control^f^	49±10	<5	<5
Scrambled^g^	<5	<5	<5
iLVS^h^	450±22	110±8	<5

^a^ CTL epitopes were derived from six proteins respectively: FTL_1916_400–410_, FTL_0966_126–133_, FTL_1708_13–23_, FTL_0283_95–104_, FTL_1101_64–74_, and FTL_1673_308–318_.

^b^ Stimulating antigens: 10 mM of the individual peptides or 10^7^ CFU/ml of formalin-inactivated LVS.

^c^ C57BL/6 mice were immunized with LVS or the DNA-PolyEp vaccine with CTL epitopes numbered (1)-(6) (see Materials and Methods).

^d^ Data represent the mean and SD of three experiments (at least two animals per experiment) that were carried out in duplicate.

^e^ The pCI vector was used to express a 186 bp DNA fragment encoding for the 1–6 epitopes (see Materials and Methods).

^f^ The peptide “ICYVSTNIM”, an identified CTL epitope in LVS not included in the DNA-PolyEp vaccine, was used as a positive control (see Materials and Methods).

^g^ Scrambled sequence of peptide (5).

^h^ LVS was inactivated by formalin (see Materials and Methods).

### Peptide Synthesis

All peptides were synthesized with 9-fluorenyl-methyloxycarbonyl (FMOC) chemistry and validated by mass spectrometry (Sigma, Israel). Peptides were adjusted to 5 mg/ml as stock solutions and stored at −20°C until used.

### Vaccination

For LVS immunizations, mice were immunized with a single dose of 10^2^ CFU LVS via the *i.n.* route after anesthesia with ketamine and xylazine. For DNA-PolyEp or pCI (used as a control) immunizations, mice were immunized 4 times at 2-week intervals with 2 µg plasmid [30], [31]. The immunizations were administered *intra-dermally* (*i.d.*) with a HELIUS gene-gun (BioRad, USA).

### Antibody Titer Analysis

Animals from each immunization group were bled prior to bacterial challenge, and the titers of anti-*F. tularensis* antibodies (IgG) in the serum samples were determined by ELISA in 96-well microtiter plates coated with 100 µl of 10^8^ CFU/ml formalin-inactivated LVS, or 10 µg of synthetic peptides. Secondary Abs goat anti-mouse IgG (Jackson ImmunoResearch Laboratories) were used in 1∶2500 dilution. Antibody titers were calculated as reciprocal geometric mean titers (GMT). Mice immunized with LVS exhibited serum-specific *F. tularensis* titers of approximately 1∶2000. The limit of detection for this assay is 1∶40.

### Splenocyte Preparation

At the designated time points, mice were euthanized, and the spleens were removed. Splenocytes were prepared in gentle MACS C-tubes (Miltenyi, Germany) according to the manufacturer’s instructions. The freshly prepared splenocytes were suspended in RPMI-1640 supplemented with 10% heat inactivated fetal calf serum, 1 mM Pen-Strep, non-essential amino acids, 2 mM L-glutamine and 2 mM sodium pyruvate. All tissue culture solutions were obtained from Biological Industries (Beit Haemek, Israel).

### IFNγ ELISPOT Assay

Single-cell suspensions of fresh splenocytes were seeded into 96-well ELISPOT plates in complete RPMI medium with 10 µM of the individual peptides or 10^7^ CFU/ml of formalin-inactivated LVS. Each peptide sample was tested in duplicate. The frequencies of the epitope-specific T lymphocytes were determined using eBioscience IFNγ ELISPOT kits with strict adherence to the manufacturer’s instructions. Throughout the analysis, the background numbers of spots in the negative control wells did not exceed five spots per well. For the determination of CD4+ or CD8+ specific T cell responses, splenocytes and 10^7^ CFU/ml of formalin-inactivated LVS were incubated with or without 10 µg/ml of rat anti-mouse CD4 (Functional Grade Purified, clone GK1.5, isotype IgG2b) or rat anti-mouse CD8 (Functional Grade Purified, clone 53–6.7, isotype IgG2a) monoclonal antibodies (eBioscience, San Diego, USA) and were tested for IFNγ production by ELISPOT assay, as mentioned above.

### In-vitro Proliferation Assay

Single-cell suspensions of fresh splenocytes were labeled with 5 µM carboxyfluorescein diacetate succinimidyl ester (CFSE) (eBioscience, San Diego, USA) for 5 minutes at room temperature and rinsed three times with RPMI+10% FCS (supplemented as described above), along with 50 µM β-mercaptoethanol and 25 mM HEPES. The labeled cells were seeded into a 24-well plate at a concentration of 10^6^ cells per plate in 1 ml of complete RPMI medium and sensitized with formalin-inactivated LVS (5×10^7^ bacterial particles per ml) in the presence of CD28- and CD49d-specific antibodies (1 µg/ml each; eBioscience, San Diego, USA) in a 37°C humidified incubator. After 48 hours of stimulation, the cells were washed, stained for CD3, CD4 and CD8 expression (eBioscience, San Diego, USA) and acquired by flow cytometry on a FACSCalibur cytometer (BD Biosciences, USA). Proliferation was analysed on gated live single lymphocytes with FlowJo software (Tri Star, USA).

### Neutrophil (CD11b^+^ Gr1^+^) Analysis

Spleens were harvested on days 1, 2 and 3 after bacterial challenge and processed as described above (splenocytes preparation). The cells were stained for CD11b (Anti-mouse CD11b, clone: M1/70, eBioscience, San Diego, USA) and Gr1 (Anti-mouse Ly-6G (Gr1), clone: RB6-8C5, eBioscience, San Diego, USA) and the data were acquired by flow cytometry on a FACSCalibur cytometer (BD Biosciences, USA). Live single cells were gated and analyzed for CD11b^+^ Gr1^+^ percentage with CellQuest software (Becton Dickinson). Neutrophils were defined by their typical light scatter, and therefore excluded from CD11b^+^ Gr1^+^ monocytes.

### Bacterial Burden Analysis

For each treatment group, three animals per time point were sacrificed to assess tissue bacterial burden. Livers and spleens were removed from each animal and homogenized in 1 ml of PBS with an Ultra-Turrax homogenizer (IKA, Germany). Ten-fold serial dilutions were carried out in PBS, 10 µl of each dilution and 100 µl of original homogenates were plated in triplicates on CHA and incubated at 37°C for 2 days. Colony counts were then taken for each sample to determine bacterial load. In addition, aliquots (100 µl) of the original homogenates were diluted in 100 µl of Triton X-100 (Sigma, Israel) and analyzed for bacterial load by RT-PCR and bacterial loads were determined according to standard curves [32].

### Statistical Analysis

Survival data were analyzed by the Kaplan-Meier log rank test using GraphPad Prism 5.01 software (San Diego, CA). A P value of <0.05 was considered statistically significant. Student’s t test (for comparison of two groups) or analysis of variance (ANOVA) (for comparison of multiple groups) was used to analyze bacterial burdens, ELISPOT tests and flow cytometric staining. All statistical tests were performed at a 5% significance level.

## Results and Discussion

### Epitope Selection and DNA-PolyEp Design

We previously described an unbiased *F. tularensis* whole genome analysis conducted in search for CD8+ T cell epitopes through a cluster based approach [26], [27]. Out of 1640 predicted and tested epitopes, 170 were confirmed to be novel CD8+ T cell epitopes [26]. All 170 responder peptides were further shown to elicit IFNγ response which could be inhibited by anti-CD8 but not by anti-CD4 antibodies [27]. Based on the relative magnitudes of the induced responses, we decided to construct a DNA-polyepitope (DNA-PolyEp) composed of the six most potent H-2K^b^/D^b^ CTL epitopes (8–11 amino acids long originating from six different proteins, Table 1 and Materials and Methods). A synthetic mini-gene (of 186 bp) coding for all six epitopes, arranged in tandem, was cloned into the pCI eukaryotic expression vector.

### DNA-PolyEp Immunization Elicits a T-cell Response to Each of the Selected Epitopes

The immunogenicity of the candidate DNA-PolyEp vaccine was evaluated in C57BL/6J mice after *intra-dermal* (*i.d.*) immunization. The ability of this vaccine to elicit a humoral response in mice was evaluated in serum samples by ELISA for specific anti-LVS and anti-peptide antibodies. None of the immunized mice showed measurable antibody response to LVS or to any of the vaccine comprising peptides (titers were below the limit of detection [<1∶40, see Materials and Methods]).

The ability of DNA-PolyEp to elicit a T cell response was evaluated by measuring the numbers of IFNγ secreting splenocytes after *in vitro* stimulation with one of the following (see Table 1): each of the individual six encoded peptides; a mix of all six peptides; an LVS-specific T cell responder peptide that was not included in the DNA-PolyEp construct (identified in the original screen [26]; “ICYVSTNIM” see Table 1); formalin inactivated LVS or a negative control peptide with a “scrambled” sequence. Splenocytes from mice that were immunized with the pCI vector DNA (which did not encode any *F. tularensis* epitope) served as an additional control for the T cell-specific response.

The response of splenocyte stimulation with each of the six peptide CTL epitopes (Table 1) demonstrated the generation of specific effector T cells in DNA-PolyEp*-*immunized mice, indicating the expression, processing and MHC presentation of each epitope. Strikingly, a mix of all six peptides, when used as stimulators in an ELISPOT assay, generated T cell responses of comparable levels in splenocytes from mice immunized with either the DNA-PolyEp vaccine or with a sub-lethal dose of LVS (Table 1). Stimulation with the negative control “scrambled sequence” peptide did not elicit any response either after LVS or following DNA-PolyEp vaccine immunization (Table 1). As expected, the CTL epitope that was not included in the DNA-PolyEp elicited a response in splenocytes from LVS-immunized mice but not in splenocytes from DNA-PolyEp immunized mice. Mice that were immunized with the pCI vector did not develop any detectable cellular immune response with all stimulants tested. Taken together, these results demonstrate that the poly-epitope expressed by the DNA vaccine is immunogenic and primes specific T cell responses to each of the six individual *F. tularensis-*encoded epitopes.

### DNA-PolyEp Immunization Specifically Primes the CD8+ T Cell Response

**Figure 1:**
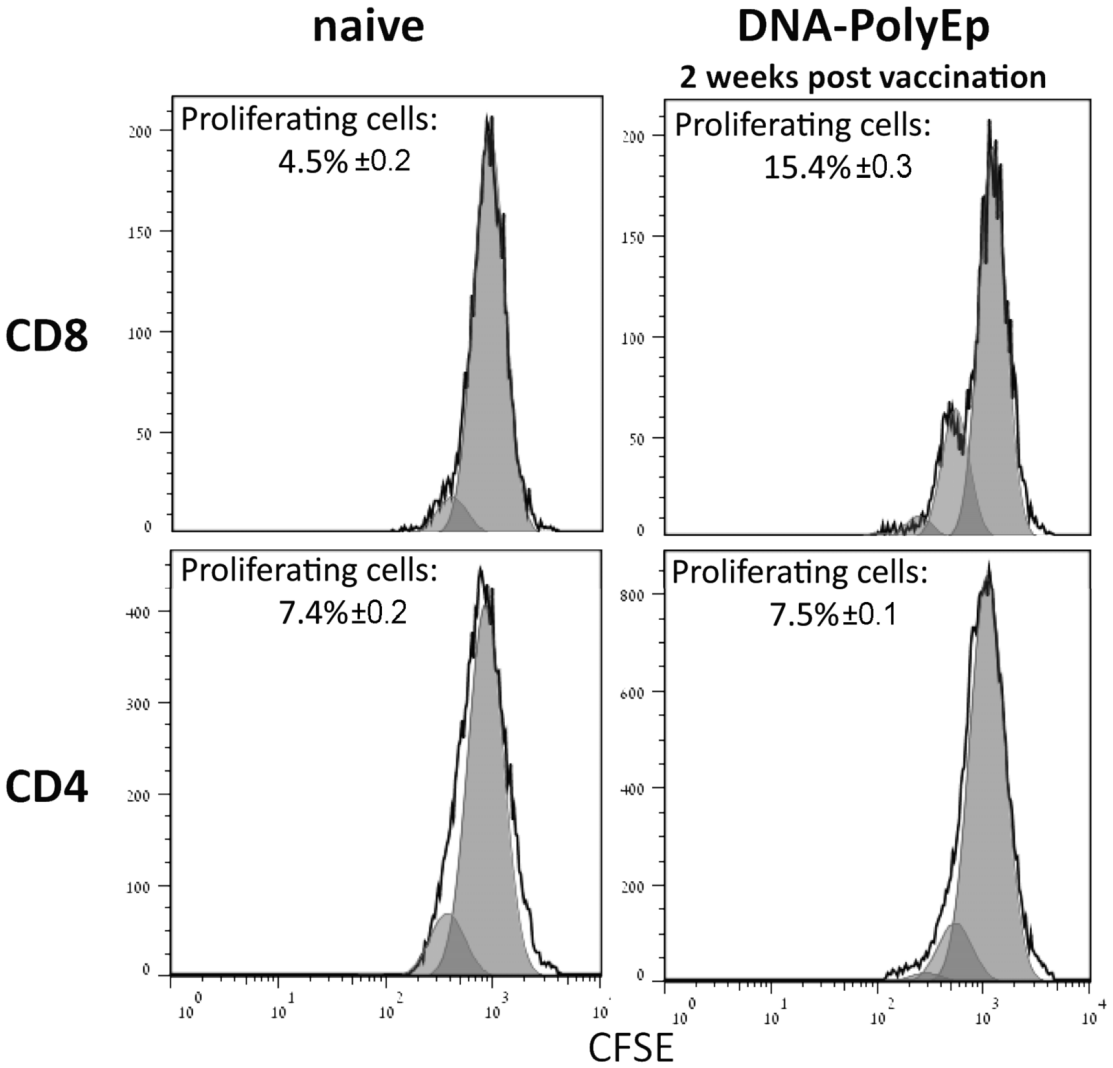
Proliferative responses of memory CD8+ and CD4+ T cells after *in vitro* stimulation. Splenocytes from DNA-PolyEp-immunized or naive mice were labeled with CFSE and stimulated for 48 hours with formalin-inactivated LVS. Stimulated cells were stained for CD4 and CD8 expression, and the proliferative responses were analyzed by flow cytometry. The percentage of splenocytes in each group represents the mean and SD of 3 individual animals from a single experiment. FACS proliferation plots are from a representative experiment.

To characterize the T cell immune response that developed after DNA-PolyEp immunization, we used an ELISPOT assay to analyze the specific populations of IFNγ secreting cells in the presence of either anti-CD8 or anti-CD4 blocking antibodies. The addition of CD4-blocking antibodies to the assay had only a minor effect (with no statistical significance) on the number of IFNγ secreting cells (Table 2), while the addition of CD8-blocking antibodies completely eliminated IFNγ secretion (limit of detection <5 SFC/1E6 cells). We note that the DNA vector (4 kbp) pCI alone did not elicit any response. To analyze the proliferative responses of memory CD4+ and CD8+ T cells, splenocytes from naive and DNA-PolyEp-immunized mice were labeled with CFSE (see Materials and Methods), stimulated *in vitro* with inactivated LVS, stained for CD4 and CD8 expression and analyzed by flow cytometry (Figure 1). In the splenocytes from DNA-immunized mice, 15.4% of the CD8+ T cells proliferated, in comparison to 4.5% in the naive mice. In contrast, no difference in proliferation was observed in the CD4+ cells from DNA-immunized mice and naïve mice (7.5%, and 7.4% respectively). The results clearly demonstrate the potential of the DNA-PolyEp vaccine to elicit an anamnestic CTL response to *F. tularensis*, a conclusion which was further supported by *in vivo* studies, as described below.

**Table 2:** Selective T cell response of vaccinated mice.

	No. of IFNγ secreting cells/10^6^ splenocytes^a^			
Immunization	Stimulation			
	iLVS^b^	iLVS+ αCD4^c^	iLVS+ αCD8^d^	No Ag^e^
DNA-multi-epitope^f^	110±8	86±9	<5	<5
pCI^f^	<5	<5	<5	<5
Naive	<5	<5	<5	<5

^a^ Number of IFNγ secreting cells/10^6^ splenocytes (splenocytes were removed 14 days following the last immunization (see Materials and Methods)). Data represents mean and SD from 3 individual animals (per group) derived from 3 independent experiments.

^b^ 10^7^ CFU/ml of formalin-inactivated LVS (see Materials and Methods).

^c^ 10 µg of anti-CD4 antibodies were added to 10^6^ splenocytes 1 hour before stimulation with formalin inactivated LVS.

^d^ 10 µg of anti-CD8 antibodies were added to 10^6^ splenocytes 1 hour before stimulation with formalin inactivated LVS.

^e^ No-Ag, samples were tested without any added antigen.

^f^ mice were immunized by gene gun. (See Materials and Methods).

### The DNA-PolyEp Vaccine Elicits a CD8 Dependent Protective Immunity Against Lethal *F. tularensis* LVS Challenge

**Figure 2:**
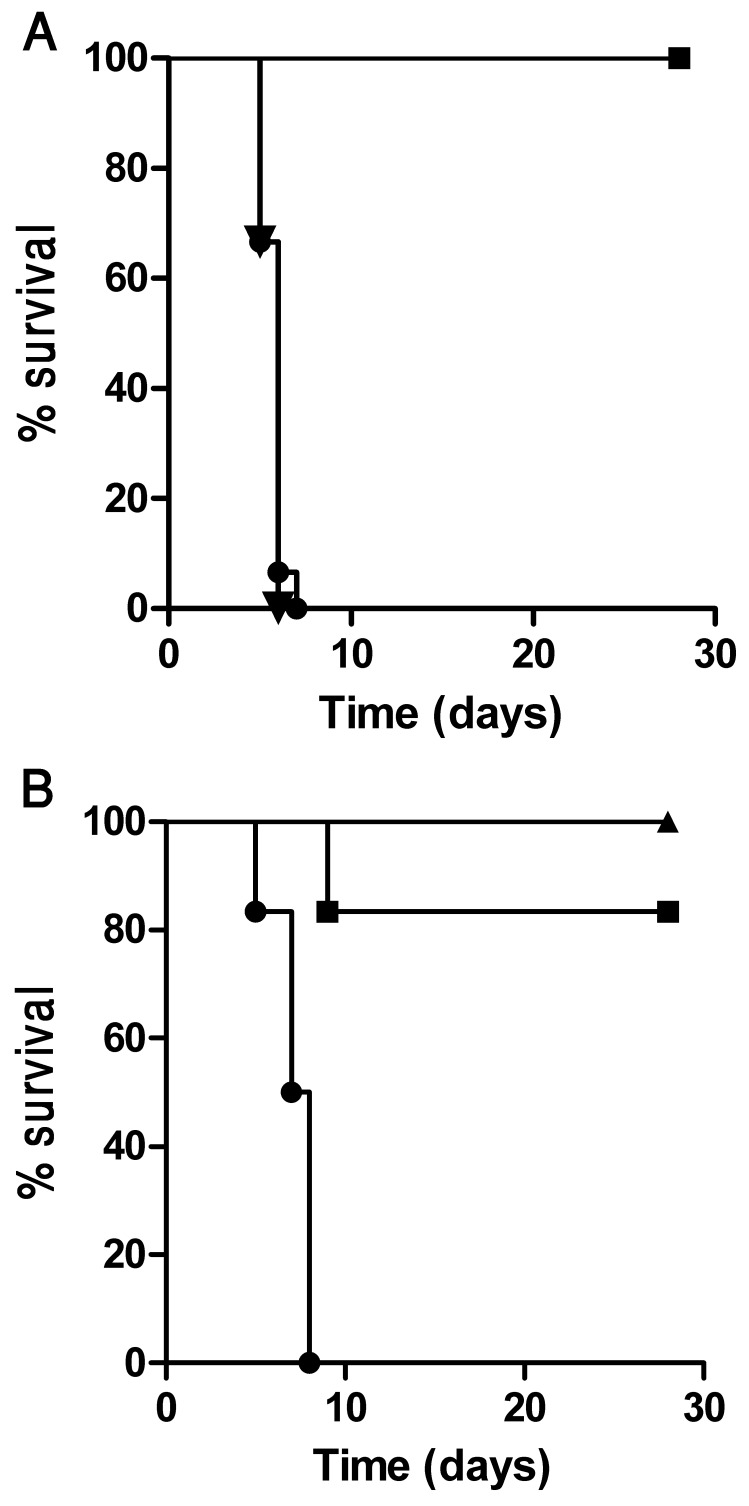
Survival of C57BL/6, CD4^−/−^ and CD8^−/−^ mice following DNA-PolyEp-immunization and *F. tularensis* LVS challenge. (A) Two weeks after completion of the immunization protocols (see Materials and Methods), mice were challenged *i.p.* with 10^3^ CFU LVS (equivalent to 10 LD_50_) and monitored for survival for 28 days. The cumulative data from 3 independent experiments (10 mice per group) are shown. Squares, DNA-PolyEp immunization; circles, pCI immunization; triangles, non-immunized mice. (B) Wild type (triangles), CD4^−/−^ (squares) and CD8^−/−^ (circles) mice (6 mice per group) were vaccinated, challenged and monitored for survival as described above.

To determine whether the CD8 immune response generated by the DNA-PolyEp vaccine could confer *in vivo* protection against LVS, the immunized mice were challenged *i.p.* with a lethal dose of 10 LD_50_ of LVS (10^3^ CFU), fourteen days after the completion of the vaccination schedule (see Materials and Methods). All of the naive non-vaccinated or the pCI vector-vaccinated mice died (MTTD of 6 days). In contrast, all of the DNA-PolyEp-vaccinated mice survived the challenge, as determined in three independent experiments (10 animals per group per experiment; Fig. 2A). Furthermore, no signs of morbidity (e.g., weight loss) were observed at any time in any of the DNA-PolyEp-vaccinated mice. In order to evaluate whether this protection is CD8+ T cells dependent, we also immunized CD8^−/−^ and CD4^−/−^ mice. All CD8-deficient mice succumbed at days 5–7 post infection, while most (5/6) of the CD4-deficient mice survived the challenge (Fig. 2B). These results provide clear support to our notion that the DNA vaccine, protects against a lethal systemic *F. tularensis* LVS infection, in a CD8 dependent manner. It should be noted that CD4^−/−^ mouse model is limited due to compensatory development of restricted CD8 cells.

**Figure 3:**
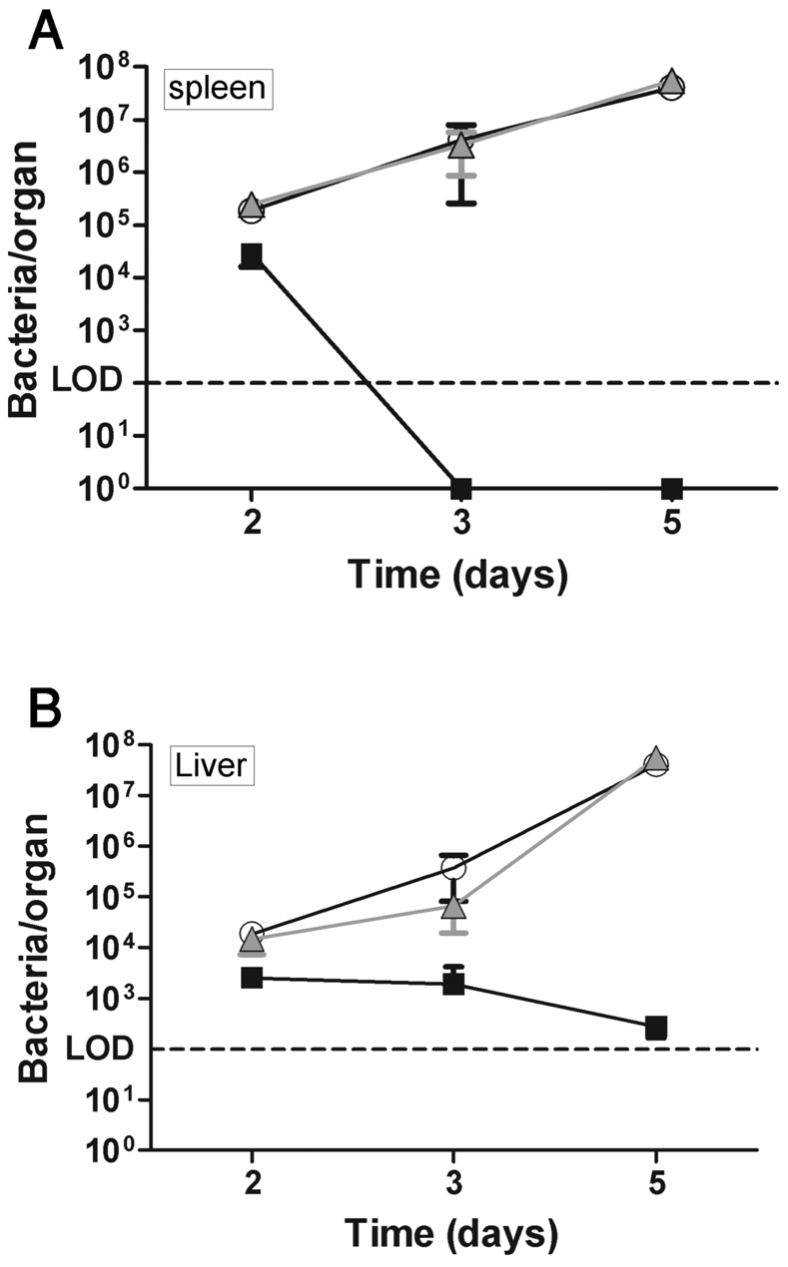
Bacterial burden in vaccinated mice after *F. tularensis* LVS challenge. Mice were vaccinated and challenged as described in figure 2. At two, three and five days post-challenge, 3 mice in each group were sacrificed, and their spleens (A) and livers (B) were removed for bacterial load inspection by CFU counts; similar results were obtained by quantitative real time-PCR. The data represent the means ± SD from 2 independent experiments. Black squares, DNA-PolyEp immunization; white circles, pCI immunization; grey triangles, non-immunized mice.

**Figure 4:**
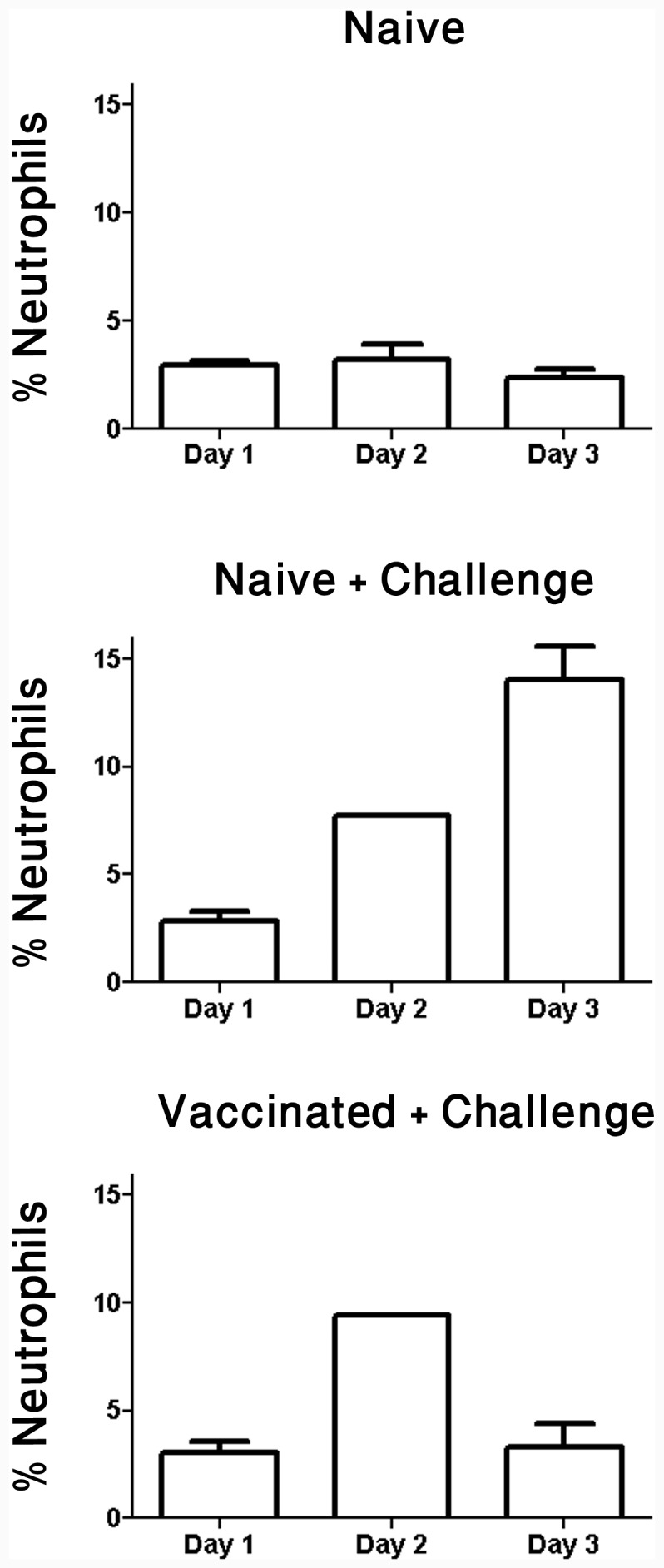
Neutrophil frequencies in the spleens of *F. tularensis* LVS challenged mice. Neutrophil (CD11b^+^ Gr1^+^) counts in the splenocytes from naive or DNA-PolyEp-vaccinated mice were analyzed by flow cytometry on three consecutive days after *i.p.* challenge with 10^3^ LVS. Bars represent the mean and SD of 3 individual animals from a single experiment.

The dynamics of the immune response against *F. tularensis* in vaccinated mice following infection was evaluated by inspecting the bacterial loads, levels of neutrophil (CD11b+Gr1+) recruitment and response of IFNγ secreting cells in selected organs (Figure 3, 4 and Table 3). Examination of the bacterial burdens (as determined by CFU or RT-PCR) demonstrated a significant 4 log_10_-fold reduction in the spleens of vaccinated mice, between days 2 and 3 post-infection. Complete clearance of the infection was observed by day 3. In contrast, and as expected, a steady increase in bacterial counts was observed in the non-immunized mice reaching approximately 10^8^ CFU/organ at five days post-infection (Fig. 3A). As could be expected, in the liver (Fig. 3B) bacterial burden in vaccinated mice appeared to be 10–20 folds lower than in the control groups between days 2 and 3 and on day 5, bacterial burden reached ∼5 log_10_-fold difference between the vaccinated and control groups. Moreover, FACS analysis revealed that both immunized and non-immunized mice showed elevated frequencies of splenic neutrophils on day 2 post-challenge (Figure 4). On day 3 post-challenge, the neutrophil (CD11b+ Gr1+) frequencies continued to rise (to approximately 15%) in the non-immunized mice. Yet, in the immunized mice, neutrophil levels returned on day 3 post challenge to levels exhibited by healthy naive mice (2–3%; Figure 4), in accordance with the observed elimination of bacteria by day 3.

**Table 3:** Selective T cell response of vaccinated mice 3 and 5 days post LVS lethal challenge.

		No. of IFNγ secreting cells/10^6^ splenocytes^a^				
		Stimulation				
	Days postchallenge^g^	iLVS^b^	iLVS+αCD4^c^	iLVS+αCD8^d^	Bacterial counts/spleen^e^	Survival^f^
Vaccinated	3	710±110	680±150	<5	<10	30/30
	5	7670±550	7020±880	<5	<10	30/30
Nonvaccinated	3	1420±160	830±70	370±30	3×10^6^	0/30
	5	6450±810	1920±120	3480±630	2×10^7^	0/30

^a^ Number of IFNγ secreting cells/10^6^ splenocytes were removed at the indicated day post challenge (see Materials and Methods). Data represents mean and SD from 3 individual animals (per group) derived from 2 independent experiments.

^b^ 10^7^ CFU/ml of formalin-inactivated LVS (see Materials and Methods).

^c^ 10 µg of anti-CD4 antibodies were added to 10^6^ splenocytes 1 hour before stimulation with formalin inactivated LVS.

^d^ 10 µg of anti-CD8 antibodies were added to 10^6^ splenocytes 1 hour before stimulation with formalin inactivated LVS.

^e^ Samples from harvested spleens were taken for the evaluation of bacterial load, which was determined by real time-PCR or CFU counts (see Fig. 3A).

^f^ Survival data are derived from 3 groups of 10 mice each at 28 days post challenge (see Fig. 2A).

^g^ Mice were challenged *i.p.* (14 days following the last immunization) with 10^3^ CFU of LVS (equivalent to 10 LD_50_)_._

In view of these remarkable results, IFNγ secretion was used to assess the presence and kinetics of an anamnestic T cell response in the immunized mice following challenge (Table 3). Comparison of data from pre-challenged (Table 2) and post-challenged (Table 3) mice revealed a fast and robust anamnestic response as manifested by a 7 fold increase (by day 3 post challenge) and almost 70 fold increase (on day 5 post challenge) in the number of IFNγ-secreting cells (110 vs. 710 and 110 vs. 7670 respectively, Table 2 and Table 3). This response was completely blocked by the addition of anti-CD8 Abs, whereas addition of anti-CD4 Abs did not exhibit any detectable effect (Table 3). Note again that all 30 immunized animals resist infection, and that the infecting bacteria are already cleared by day 3. In contrast, in naïve (non-immunized) mice the high bacterial load following challenge (3×10^6^ on day 3 and 2×10^7^ on day 5) induced an inflammatory response which includes mainly NK and also T cell response which is mediated by both CD4+ and CD8+ and all animals eventually died (Table 3).

We may conclude from the results described above (Fig. 2, 3, 4 and Tables 2 and 3) that the efficacy of the DNA-PolyEp LVS vaccine is realized through a potent and rapid *F. tularensis* LVS-specific, anamnestic CD8+ T cell response that permits the effective clearance of the bacterial pathogen by the wild type mice, leading to a full protection against (10LD_50_ of i.p challenge) without any sign of disease (in 30 of 30 animal used in this study). The absence of any protection in CD8^−/−^ mice, demonstrate that this protective immunity is entirely dependent on CD8+ T cells.

**Figure 5:**
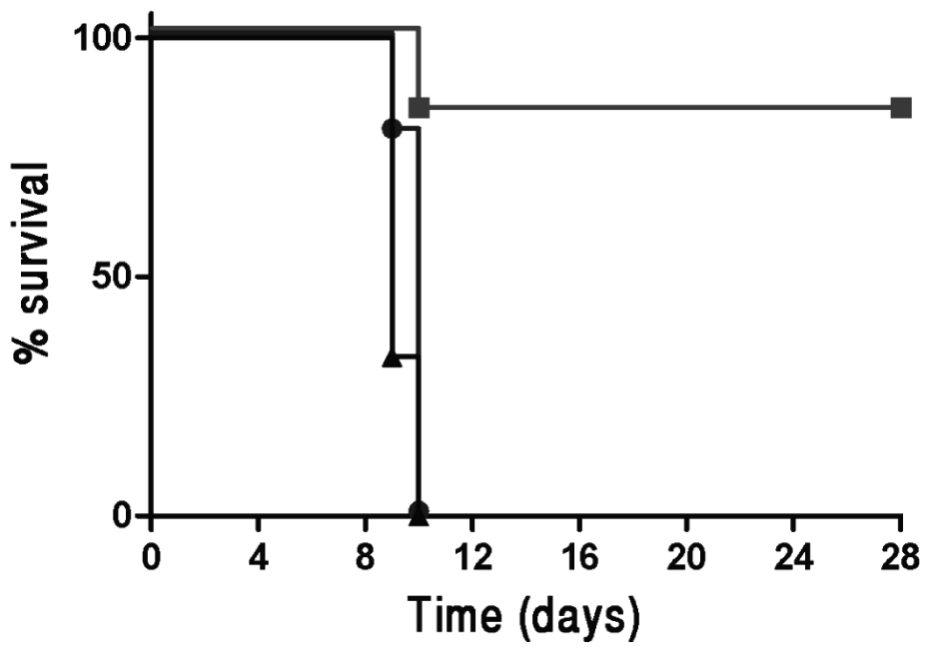
Survival after lethal inhalational *F. tularensis* LVS challenge. Two weeks after the completion of the immunization schedule (see Materials and Methods), 10 mice in each vaccine group were challenged *i.n.* with 10^4^ CFU LVS (equivalent to 10 LD_50_) and monitored for survival for 28 days. squares, DNA-PolyEP immunization; circles, pCI immunization; triangles, non-immunized mice.

We have demonstrated that systemic (*i.d.*) immunization confers solid protection against lethal systemic infection (*i.p.*). However, it is well established that the most severe manifestations of the disease result from inhalation of the bacterial agent. Accordingly, we tested whether *i.d.* immunization with DNA-PolyEp could confer protection against airway infection with *F. tularensis*. To this end, the DNA-PolyEp *i.d.*-vaccinated mice were *i.n.* challenged with 10 LD_50_ (10^4^ CFU) LVS fourteen days after completion of the vaccination schedule. While all of the naive non-vaccinated and pCI vector-vaccinated mice died, 80% (8/10) of the DNA-PolyEp-vaccinated animals survived the challenge (Fig. 5). However, in contrast to the *i.p.* challenged mice, signs of morbidity (weight loss) were observed in all *i.n.* infected animals.

Since all of the epitopes included in the DNA-PolyEp are shared by both *F. tularensis holarctica* (LVS) and the highly virulent *F. tularensis tularensis* Schu S4 strain, it was of interest to determine whether the DNA-PolyEp construct can also protect animals from a lethal Schu S4 *i.p.* challenge. It should be noted however, that vaccination with live attenuated LVS provides effective protection against its homologous *F. tularensis holarctica* strain but has limited ability to protect against the fully virulent heterologous *F. tularensis tularensis* Schu S4 strain ([12], [18], [33] and our unpublished results). Moreover, functionality of both CD4+ and CD8+ T cells in LVS-vaccinated mice is required in order to withstand a challenge of the virulent Schu S4 *F. tularensis* [12], [13], [34]. Thus it was not too surprising, that the DNA-PolyEp failed to protect mice from a 10LD_50_ *i.p* Schu S4 challenge (data not shown).

There is clearly a need for improvement of the poly epitope-based DNA vaccine which could possibly be achieved by various adjuvants and/or inclusion of other Schu S4 specific potent CD8/CD4 epitopes, as well as other modalities of vaccination.

To date, there have only been a few documented examples in which CTL-inducing vaccines have been shown to confer protection against intracellular bacteria. Most of these studies relied on the prior knowledge of specific immunogenic proteins or the selection of proteins that were known to be involved in virulence. Examples include the identification of immunodominant CD8 epitopes from *Listeria monocytogenes* virulence factors [35], [36] such as LLO_91–99_, which was shown to mediate rapid bacterial clearance [35], [37]–[39]. Another example is the bacterial immunodominant CD8 epitope located in YopE, a well-known virulence factor of *Yersinia pestis*. Immunization with this peptide protected mice against pulmonary infections with an attenuated variant of *Y. pestis* [40], [41]. Several CD8 epitopes that were identified in *Chlamydia pneumonia* by an immunoinformatic screen of 35 proteins [42] which were assumed to reach the host cytoplasm and were thus used to construct a DNA mini-gene. However, although this vaccine was effective in promoting bacterial clearance, it provided partial protection against *C. pneumonia* infections [43]. A previous attempt to generate a T cell vaccine based on poly-CD4 epitopes of *F. tularensis* was shown to provide some protection against *intra-tracheal* challenge with LVS in transgenic mice (class II HLA) [25].

In conclusion, we have shown that protection against lethal *F. tularensis* LVS challenge can be achieved by vaccination with few selected CD8+ T cell epitopes without detectable contribution of CD4+ T cells. Vaccine efficacy may be attributed to the generation of a rapid and potent anamnestic *F. tularensis* CD8+ specific T cell response. Furthermore, the epitopes that conferred this protection were selected according to their locations in hotspots/clusters for MHC-I binding, rather than by the classical criteria of predicted affinity values or prior information about the source proteins. To the best of our knowledge, this study represents the first example for the design of an efficacious CD8+ T cell-mediated vaccine against lethal infection of an intracellular bacterial pathogen, based on an unbiased whole-genome immunoinformatic CTL epitope screen. Our approach provides a proof-of-principle for selecting and generating a multi-epitpoe CD8 T cell-stimulating vaccine against a model intracellular bacterium.
